# The Practical Medical Series of Year Books

**Published:** 1902-04

**Authors:** 


THE PRACTICAL MEDICAL SERIES OF YEAR BOOKS. Ten volumes. Issued monthly, under the general editorial charge of GUSTAVUS P. HEAD, M. D., Professor of Laryngology and Rhinology in the Chicago Post-Graduate Medical School. Vol. II. General Surgery. Edited by John B. Murphy, M. D., Professor of Surgery, Northwestern University Medical School. November, 1901. Pp. 515. Chicago: The Year Book Publishers, 40 Dearborn Street. [Cloth, $2.00.]

The present volume, dated November, 1901, is one of the Practical Medicine Series of Year Books. Of these there are ten covering the entire field of medicine and surgery. The purpose of this work is to present an epitomé of the progress of surgery for the preceding year. The editors and authors evidently have an accurate conception of what a work of this kind should be. The work is published at a low figure and is cheaply printed. A number of illustrations are introduced, the quality of many of which is seriously injured by being half-tones of previous half-tones. As a result the introduction of two series of cross lines into the illustrations gives them a very unsatisfactory appearance and seriously interferes with the definition. It would be wiser if in these cases the illustrations were re-drawn, or if attempts were made to obtain the originals from the authors for purposes of reproduction.

The text of the work fairly represents the advance of surgery for the past year and the editor has shown rare good judgment in the selection and presentation of his material. The work has the advantage of not attempting to be too voluminous and at the price published should find its way to the desk of many surgeons. H.R.G.

